# (1′*S*)-1′-Acetoxyeugenol Acetate Enhances Glucose-Stimulated Insulin Secretion

**DOI:** 10.3390/plants12030579

**Published:** 2023-01-28

**Authors:** Dahae Lee, So-Ri Son, Yutong Qi, Ki Sung Kang, Dae Sik Jang

**Affiliations:** 1 College of Korean Medicine, Gachon University, Seongnam 13120, Republic of Korea; 2 Department of Biomedical and Pharmaceutical Sciences, Graduate School, Kyung Hee University, Seoul 02447, Republic of Korea; 3 Department of Life and Nanopharmaceutical Sciences, Graduate School, Kyung Hee University, Seoul 02447, Republic of Korea; 4 Department of Pharmaceutical Science, College of Pharmacy, Kyung Hee University, Seoul 02447, Republic of Korea

**Keywords:** *Alpinia galanga*, (1′*S*)-1′-acetoxyeugenol acetate, Insulin, α-glucosidase, PDX-1

## Abstract

*Alpinia galanga* have been widely used as spice or traditional medicine in East Asia, commonly known as Thai ginger. In the present study, seven major phenylpropanoids, (±)-1′-hydoxychavicol acetate (**1**; HCA), (1′*S*)-1′-acetoxychavicol acetate (**2**; ACA), (1′*S*)-1′-acetoxyeugenol acetate (**3**; AEA), eugenyl acetate (**4**), *trans*-*p*-coumaraldehyde (**5**), *trans*-*p*-acetoxycinnamyl alcohol (**6**), and *trans*-*p*-coumaryl diacetate (**7**), were isolated from the 95% EtOH and hot water extracts of the rhizomes of *A*. *galanga* by chromatographic method. Phenylpropanoids **1**–**7** were evaluated for glucose-stimulated insulin secretion (GSIS) effect and α-glucosidase inhibitory activity. Phenylpropanoids **1**–**4** increase GSIS effect without cytotoxicity in rat INS-1 pancreatic β-cells. In addition, INS-1 cells were treated with AEA (**3**) to determine a plausible mechanism of β-cell function and insulin secretion through determining the activation of insulin receptor substrate-2 (IRS-2), phosphatidylinositol 3-kinase (PI3K), Akt, and pancreatic and duodenal homeobox-1 (PDX-1). Upon treatment with AEA (**3**), INS-1 cells showed an increase in these protein expressions. Meanwhile, AEA (**3**) exhibited α-glucosidase inhibitory activity. On the basis of the above findings, we suggest AEA (**3**) as a potential antidiabetic agent.

## 1. Introduction

Diabetes is one of the world’s fastest-growing chronic metabolic disorders, characterized by hyperglycemia and impaired glucose metabolism. The impaired insulin action in the liver, muscle, and adipose tissue, or insufficient secretion of insulin from the pancreatic β-cells, or both, contribute to the onset and progression of diabetes [1]. Type 2 diabetes (T2D) makes up about 90% of patients with diabetes. Maintaining a balance between insulin secretion and absorption of blood sugar is necessary to prevent or delay T2D [2].

The major classes of pharmacological agents for diabetes are largely divided into three types: insulin secretagogues, insulin sensitizers, and carbohydrate digesting enzyme inhibitors [3]. Insulin secretagogues decrease blood glucose by stimulating the insulin secretion, and include sulfonylureas (glibenclamide, gliclazide, etc.), glinides (repaglinide, nateglinide, mitiglinide, etc.), and incretin-related drugs (dipeptidyl peptidase-4 inhibitors and glucagon-like peptide-1 receptor agonists) [4]. Insulin sensitizers enhance insulin sensitivity in peripheral tissues and include thiazolidinediones (pioglitazone, rosiglitazone, etc.) and biguanides (metformin, phenformin, etc.) [5]. A carbohydrate digesting enzyme inhibitor, such as acarbose, inhibits intestinal α-glucosidase activity responsible for the metabolism of carbohydrates [6]. These drugs have been reported to be related to serious side effects. For example, gliclazide induces dizziness and abdominal pain, while acarbose causes abdominal pain and diarrhea [7]. In traditional medicines for diabetes, natural products such as *Panax ginseng*, *Opuntia ficus-indica*, and *Momordica charantia* have long been used in patients to lower blood glucose [8]. A biguanide, metformin, comes from a guanidine-rich plant, *Galega officinalis* [9]. These natural products have played an important role in diabetes management, but there is still a need for research to discover more effective natural products with decreased side effects.

*Alpinia galanga* (L.) Willd. (family Zingiberaceae) have been widely used as a spice or traditional medicine in East Asia, commonly known as Thai ginger. Recently, scientific research has demonstrated that *A*. *galanga* is a source of compounds with strong pharmacological activity, including anti-cancer, anti-obesity, neuroprotection, anti-allergy, anti-fungal, and anti-inflammatory [10,11,12,13,14,15]. 1′-Acetoxychaviol acetate (ACA) is a major constituent in *A*. *galanga* with significant pharmacological properties in multiple disease models [16]. ACA has a potent anti-cancer effect by stimulating the apoptotic signaling pathway or generating reactive oxygen species (ROS) in diverse cancer cell lines, including human colorectal cancer, leukemia, lung adenocarcinoma, and breast cancer cell lines [17]. In addition to its anti-cancer properties, ACA is also active in anti-obesity, anti-microbial, and gastroprotective models [17]. However, few studies have been conducted on other main phenylpropanoids than ACA, and the structure-activity relationship of phenylpropanoids in *A*. *galanga* has received relatively little attention. In order to explore other pharmacological effects of phenylpropanoids in *A*. *galanga*, seven major phenylpropanoids (**1**–**7**) were isolated from the hot water extract of *A*. *galanga* rhizomes by chromatographic method. *A. galanga* has been reported to have antidiabetic effects by regulating pancreatic β-cell regeneration and blood sugar levels in diabetic rats [18]. However, there are few reports on *A. galanga* responsible for increasing insulin secretion and inhibiting α-glucosidase activity [18,19], which can ultimately prevent or delay diabetes. Thus, the objective of the present study was to evaluate the insulin secretion effects of phenylpropanoids (**1**–**7**) isolated from the hot water extract of *A*. *galanga*, and their α-glucosidase inhibitory effects in rat INS-1 pancreatic β-cells. Furthermore, a plausible mechanism of β-cell function and insulin secretion was evaluated. Pancreatic and duodenal homeobox-1 (PDX-1), a transcription factor, is related the to function and survival of pancreatic β cells [20]. PDX-1 is regulated by a wide-range of upstream signaling, including insulin receptor substrate-2 (IRS-2) [21], phosphatidylinositol 3-kinase (PI3K) [22], and Akt [23]. To determine the expressions of PDX-1, IRS-2, PI3K, and Akt, a Western blot was performed.

## 2. Results

### 2.1. Isolation and Identification of Phenylpropanoids **1**–**7** from the Rhizomes of A. galanga

In our previous work, we isolated three major phenylpropanoids (compounds **2**–**4**) from the 95% EtOH extract of *A*. *galanga* rhizomes [24]. In the present study, an additional four major phenylpropanoids (compounds **1** and **5**–**7**) were isolated from the hot water extract of *A*. *galanga* rhizomes by chromatographic method. The plain structures of compounds were confirmed by interpretation of 1D-NMR spectroscopic data (Figures S1–S7) and by comparison with previously reported data [25,26,27,28,29,30,31].

In order to determine the absolute configuration of compounds **1**–**3**, specific optical rotation values of **1**–**3** were measured and compared to published values [26,28]. Compound **1** is a racemic mixture, since its average specific rotation was close to zero [−0.4° (c 0.1, EtOH)] (Table S1). Compounds **2** and **3** exhibited negative values of specific rotation [−50.2° (*c* 0.1, EtOH) and −16.6° (c 0.21, EtOH), respectively] (Table S1). According to the previous research, the optical rotation value for the 1′*S* configuration of compound **2** is negative, while the value for the 1′*R* configuration is positive [26]. Additionally, the 1′*S* configuration of compound **3** has also been reported to have a negative value [28]. Thus, the absolute configuration of compounds **2** and **3** was determined as 1′*S*.

**Figure 1:**
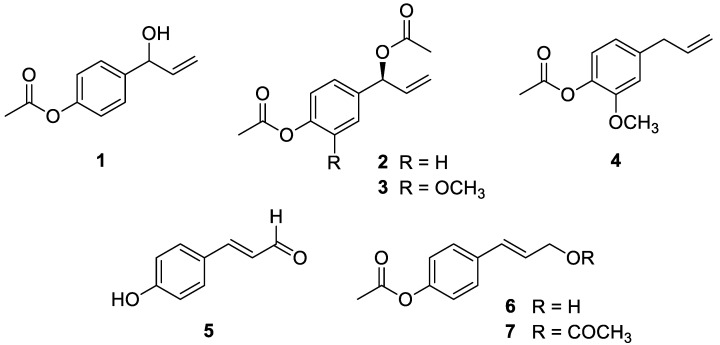
Phenylpropanoids **1**–**7** isolated from the rhizomes of *A. galanga*.

As a result, compounds **1**–**7** were identified as: (±)-1′-hydoxychavicol acetate (**1**; HCA) [25,26], (1′*S*)-1′-acetoxychavicol acetate (**2**; ACA) [26,27], (1′*S*)-1′-acetoxyeugenol acetate (**3**; AEA) [24,28], eugenyl acetate (**4**) [29], *trans*-*p*-coumaraldehyde (**5**) [30], *trans*-*p*-acetoxycinnamyl alcohol (**6**) [31], and *trans*-*p*-coumaryl diacetate (**7**) [28] (Figure 1).

### 2.2. Effects of Phenylpropanoids **1**–**7** on Glucose-Stimulated Insulin Secretion

**Figure 2:**
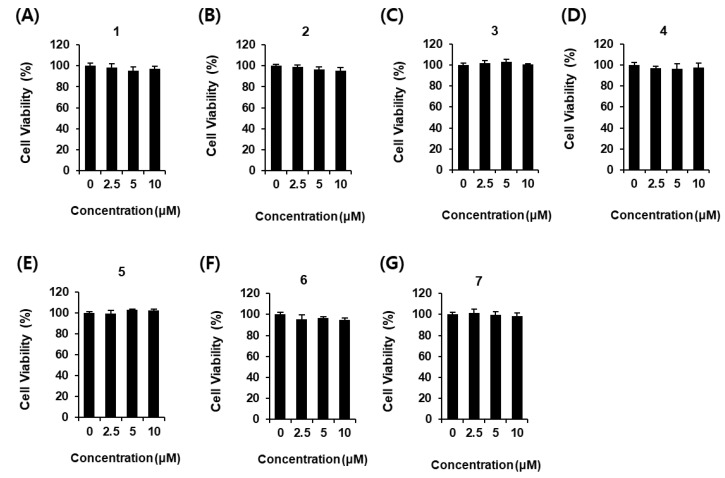
Effects of phenylpropanoids **1**–**7** on the viability of INS-1 cells. Effect of (**A**–**G**) compounds **1**–**7** compared with the control (0 μM) on the viability of INS-1 cells for 24 h by MTT assay (*n* = 3 independent experiments, *p* < 0.05, Kruskal–Wallis nonparametric test). Data represent the mean ± SEM.

**Figure 3:**
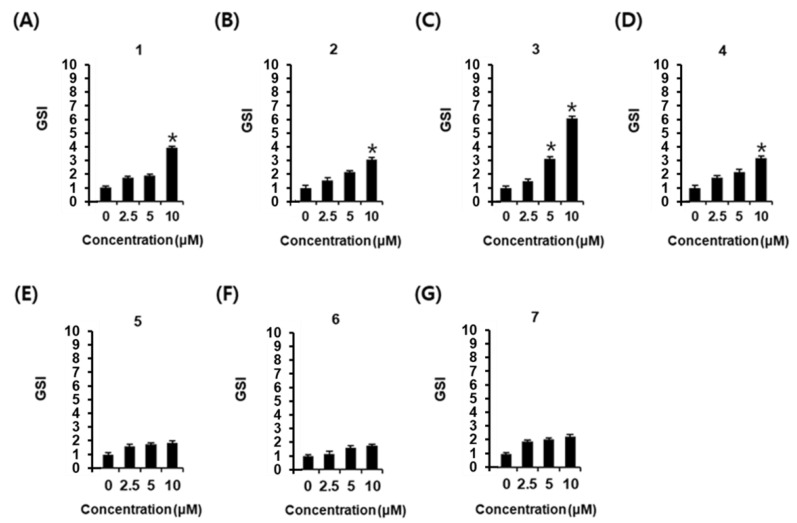
Effects of phenylpropanoids **1**–**7** on glucose-stimulated insulin secretion in INS-1 cells. Effect of compounds **1**–**7** (**A**–**G**) compared with the control (0 μM) on GSIS in INS-1 cells for 1 h by insulin secretion assay. The fold change in GSIS was expressed in terms of the glucose stimulated index (GSI, 16.7 mM/2.8 mM glucose for 1 h). (*n* = 3 independent experiments, * *p* < 0.05, Kruskal–Wallis nonparametric test). Data represent the mean ± SEM.

Phenylpropanoids **1**–**7** were tested for their effect on cell viability to select the non-toxic concentration to be used in the glucose-stimulated insulin secretion assay, and did not show any toxicity at any concentration (Figure 2). As shown in Figure 3, phenylpropanoids **1**–**4** increased glucose-stimulated insulin secretion (GSIS). GSIS was expressed as the GSI. GSI values were: 3.95 ± 0.11 for HCA (**1**) at a concentration of 10 μM (Figure 3A); 3.12 ± 0.11 for ACA (**2**) at a concentration of 10 μM (Figure 3B); 3.17 ± 0.14 and 6.16 ± 0.14 for AEA (**3**) at concentrations of 5 and 10 μM (Figure 3C); 3.95 ± 0.11 for eugenyl acetate (**4**) at a concentration of 10 μM (Figure 3D). Among these phenylpropanoids, the GSI of AEA (**3**) was the highest, and it was selected as the subject of further mechanistic studies.

### 2.3. Effects of AEA (**3**) on the Protein Expression of P-IRS-2, IRS-2, P-PI3K, PI3K, P-Akt (Ser473), Akt, and PDX-1

**Figure 4:**
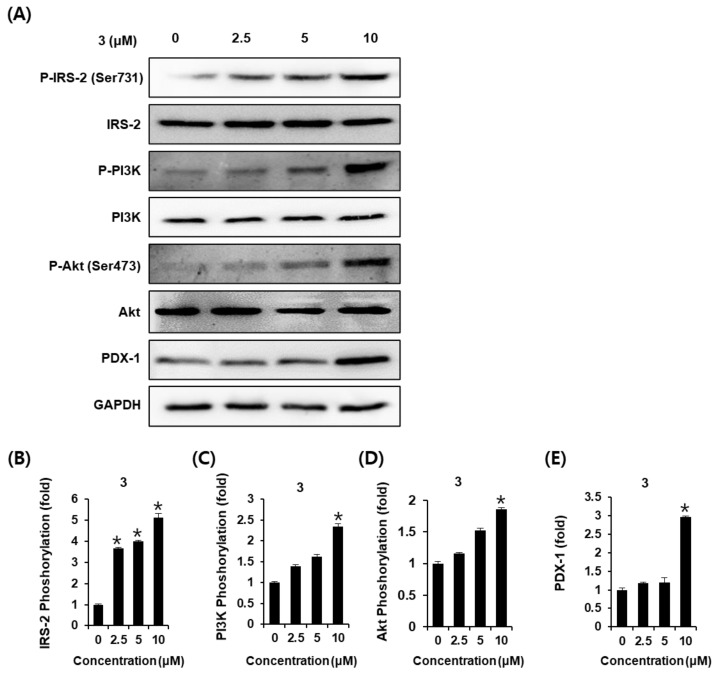
Effects of AEA (**3**) on the protein expression levels of phospho-insulin receptor substrate-2 (P-IRS-2) (Ser731), IRS-2, phospho-phosphatidylinositol 3-kinase (P-PI3K), PI3K, phospho-Akt (P-Akt) (Ser473), and Akt, and pancreatic and duodenal homeobox-1 (PDX-1) in INS-1 cells. (**A**) Protein expression levels of P-IRS-2 (Ser731), IRS-2, P-PI3K, PI3K, P-Akt (Ser473), Akt, pancreatic and duodenal homeobox-1 (PDX-1), and glycer-aldehyde 3-phosphate dehydrogenase (GAPDH) in INS-1 cells treated or untreated with 2.5 μM, 5 μM, and 10 μM AEA (**3**) for 24 h. (**B**–**E**) The bar graph presents the densitometric quantification of Western blot bands (*n* = 3 independent experiments, * *p* < 0.05, Kruskal–Wallis nonparametric test). Data represent the mean ± SEM.

To explore the underlying influence of AEA (**3**) on glucose-stimulated insulin secretion in INS-1 cells, the expression of proteins related to pancreatic β-cell metabolism was analyzed. As shown in Figure 4A, 10 μM of AEA (**3**) increased the relative abundances of P-IRS-2 (Ser731), P-PI3K, P-Akt (Ser473), and PDX-1 proteins. The bar graphs illustrate the ratio of P-IRS-2 (Ser731), P-PI3K, P-Akt (Ser473), and PDX-1 expression normalized to their corresponding GAPDH expression (Figure 4B–E). These results suggest that the effect of AEA (**3**) enhances the expression of proteins related to pancreatic β-cell metabolism.

### 2.4. Effects of AEA (**3**) on α-Glucosidase Inhibitory Activity

**Figure 5:**
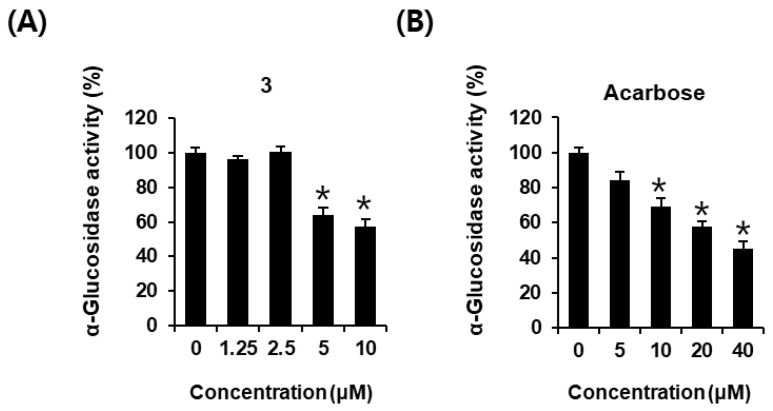
α-Glucosidase-inhibitory activities of (**A**) AEA (**3**) and (**B**) acarbose (positive control) compared with that of the control (0 μM) by α-glucosidase-inhibitory activity assay (*n* = 3 independent experiments, * *p* < 0.05, Kruskal–Wallis nonparametric test). Data are expressed as the mean ± SEM.

As shown in Figure 5A, α-glucosidase activity was 64.27 ± 0.43% and 57.43 ± 0.39% after incubation with AEA (**3**) at 5 and 10 μM, respectively, compared with that of the control (0 μM). AEA (**3**) were more effective than acarbose (positive control) of the same concentration. α-Glucosidase activity was 69.14 ± 4.89%, 57.76 ± 2.96%, and 45.03 ± 4.43% after incubation with acarbose at 10, 20, and 40 μM, respectively, compared with that of the control (0 μM) (Figure 5B). This result showed that AEA (**3**) could be a potential α-glucosidase inhibitor.

## 3. Discussion

Type-1 diabetes is characterized by insulin deficiency due to the destruction of pancreatic β cells, while T2D is associated with a gradual loss of insulin secretion [32]. Therefore, impairment of GSIS is known to be a risk factor for developing T2D, while, at the same time, improved GSIS might be a strategy for the discovery of a potential agent to treat T2D. In the present study, seven major phenylpropanoids were isolated from the 95% EtOH and hot water extracts of *A. galanga*. The phenylpropanoids **1**–**7** were identified as HCA (**1**), ACA (**2**), AEA (**3**), eugenyl acetate (**4**), *trans*-*p*-coumaraldehyde (**5**), *trans*-*p*-acetoxycinnamyl alcohol (**6**), and *trans*-*p*-coumaryl diacetate (**7**). Among the isolated phenylpropanoids, phenylpropanoids **1**–**4** increased GSIS. *p*-Coumaraldehyde (**5**), a major component of cinnamon and *A*. *galanga*, induces apoptosis in L-60 and U937 human leukemic cells via mitochondrial and endoplasmic reticulum stress pathways [33]. The antimicrobial activity of *p*-coumaryl diacetate (**7**) against methicillin-resistant *Staphylococcus aureus* (MRSA) has been reported [34]. The ACA (**2**) was also enacted because it has a significant anti-bacterial effect on MRSA [35]. ACA also possesses anti-inflammatory and anti-cancer properties [36,37]. Despite their pharmacological effects, compounds **5**–**7** did not have significant effects on GSIS in this study. As far as we know, no in vitro study has yet assessed the effect of HCA (**1**), ACA (**2**), AEA (**3**), and eugenyl acetate (**4**) on GSIS effect. Thus, these beneficial results may be of great importance for the discovery of a potential agent to treat T2D.

Insulin secretion and pancreatic β-cell function are tightly coupled and regulated through a network of multiple interacting transcription factors [38,39]. It has been reported that the role of PPAR-γ agonists in pancreatic β cells is related to improved GSIS and decreased serum proinsulin to insulin ratio. An increase in serum proinsulin to insulin ratio is an indicator of the impaired secretory response of β cells [40]. PDX-1 is a frequently mentioned transcription factor involved in the function of pancreatic β cells [41]. In animal studies using mice, a complete lack of PDX-1 impairs GSIS [42]. Expression of the PDX-1 within islet β-cells leads to GSIS through membrane-mediated insulin-containing vesicular exocytosis [43]. An inadequate β-cell mass of 50% or less is known to induce impaired GSIS [44]. Insulin receptor substrate proteins (IRS-1 and IRS-2) have been reported to maintain the normal pancreatic β-cell function [45]. IRS-1 is involved in the regulation of insulin production in pancreatic β-cells, while IRS-2 controls the pancreatic β-cell mass, which decides the limit of insulin production [21]. PI3K and Akt are important downstream signaling molecules for IRS-2. Activation of the PI3K/Akt pathway contributes to the adequate mass of functional pancreatic β cells, as well as the nuclear translocation of PDX-1 [46]. Thus, the current study examined the changes in protein expression of P-IRS-2 (Ser731), P-PI3K, P-Akt (Ser473), and PDX-1 in INS-1 cells treated with AEA (3). It was observed that the protein expression levels of P-IRS-2 (Ser731), P-PI3K, P-Akt (Ser473), and PDX-1 were increased by treatment with AEA (**3**) at 10 μM compared to the untreated controls. These results enhance the understanding of the underlying mechanisms of AEA (**3**) on the amelioration of GSIS.

Since the inhibition of α-glucosidase, a carbohydrate hydrolyzing enzyme, lowers blood glucose levels by delaying the digestion of carbohydrates, α-glucosidase inhibitors such as acarbose and miglitol could help to treat T2D [47]. Although many natural products have already been reported as sources of α-glucosidase inhibitors [6], researching natural products with great structural diversity still offers an attractive strategy for finding α-glucosidase inhibitors. In the present study, AEA (**3**) inhibited the α-glucosidase activity. AEA (**3**) was more effective than acarbose (positive control) at the same concentration. This result shows that AEA (**3**) could partly replace acarbose as a potential α-glucosidase inhibitor. The next step in our research would be to inspect the side effects of AEA (**3**) to compare them with those reported for acarbose—meteorism and abdominal distention [48].

## 4. Materials and Methods

### 4.1. Isolation of Phenylpropanoids **1**–**7** from A. galanga

The rhizomes of *A*. *galanga* were supplied from GNP BIO Co., LTD (Seoul, South Korea) and identified by Prof. Dae Sik Jang. A voucher specimen (ALGA-2018) has been kept in the Natural Product Medicine Laboratory of Kyung Hee University. The grinded rhizomes of *A*. *galanga* (100.0 g) were extracted twice with hot water under reflux condition for 2 h to produce a hot water extract. The extract (22.8 g) was dissolved in water and suspended in the same volume of EtOAc for the production of an EtOAc-soluble fraction (ALGAE, 944.3 mg). ALGAE was fractionated using silica gel column chromatography (CC; 230–400 mesh; *ϕ* 3.6 × 27.0 cm; *n*-hexane: EtOAc = 8.5:1.5 to 5:5, *v*/*v*) to generate 25 fractions (ALGAE1~25). ALGAE14 and ALGAE21 were identified as compounds **1** (20.7 mg) and **7** (11.5 mg), respectively. Compound **5** (4.9 mg) was obtained by separating ALGAE23 using flash CC with an ODS cartridge (13.0 g) with a gradient solvent system (acetonitrile:H_2_O = 2:8 to 3:7, *v*/*v*). Compound **6** (79.6 mg) was obtained from ALGAE22 by a flash CC system equipped with an ODS cartridge (13.0 g; acetonitrile:H_2_O = 2.8:7.2 to 3.8:6.2, *v*/*v*). Compounds **2** (3.98 g), **3** (36.8mg), and **4** (14.6 mg) were isolated by 95% EtOH extraction with an isolation procedure that has been described in previously published methods [19].

### 4.2. Cell Culture

Rat INS-1 pancreatic β-cells (Biohermes, Shanghai, China) were grown in an RPMI-1640 (Cellgro, Manassas, VA, USA) containing 10% fetal bovine serum (FBS), 1% penicillin/streptomycin (Invitrogen Co., Grand Island, NY, USA), 11 mM D-glucose, 2 mM L-glutamine, 10 mM HEPES, 0.05 mM 2-mercaptoethanol, and 1 mM sodium pyruvate under 5% CO_2_ and 95% air atmosphere with saturated humidity.

### 4.3. Cell Viability Assay

INS-1 cells were seeded at a density of 1 × 10^4^ cells per well in 96-well plates for 24 h. INS-1 cells were treated with phenylpropanoids **1**–**7** at concentrations of 2.5, 5, and 10 μM for 24 h to assess their non-toxic dose ranges. The samples of phenylpropanoids **1**–**7** were first dissolved in DMSO at 100 mM concentrations, and then diluted to the desired concentrations in RPMI-1640 medium supplemented with 1% penicillin/streptomycin and 10% FBS. The control (0 μM) solvent used same media. Cell viability was identified according to the manual of the Ez-Cytox cell viability detection kit (Daeil Lab Service Co., Seoul, Korea). Ez-Cytox reagent, at the indicated concentrations, was added per well under light-limited conditions and its absorbance at 450 nm was measured using a PowerWave XS microplate reader (Bio-Tek Instruments, Winooski, VT, USA).

### 4.4. GSIS Assay

INS-1 cells were seeded at a density of 4 × 10^5^ cells per well in 12-well plates for 24 h. INS-1 cells were carefully washed twice with Krebs-Ringer bicarbonate HEPES buffer (KRBB, 4.8 mM KCl, 129 mM NaCl, 1.2 mM KH_2_PO_4_, 1.2 mM MgSO_4_, 2.5 mM CaCl_2_, 10 mM HEPES, 5 mM NaHCO_3_, and 0.1% bovine serum albumin (BSA), pH 7.4). To induce starvation, cells were incubated with fresh KRBB for 2 h, and treated with phenylpropanoids **1**–**7** at concentrations 2.5, 5, and 10 μM. After treatment for 2 h, INS-1 cells were stimulated with normal (2.8 mM) and high (16.7 mM) glucose, respectively, for 1 h. GSIS was measured according to the manual of the rat insulin ELISA kit (Gentaur, Shibayagi Co. Ltd., Shibukawa, Gunma, Japan). The fold change in GSIS was expressed in terms of the glucose-stimulated index (GSI, 16.7 mM/2.8 mM glucose for 1h).

### 4.5. Western Blot Analysis

INS-1 cells were seeded at a density of 8 × 10^5^ cells per well in 6-well plates for 24 h. Then, the cells were treated with AEA (**3**) at concentrations of 2.5, 5, and 10 μM for 24 h. The expression of proteins including phospho-insulin receptor substrate-2 (P-IRS-2) (Ser731), IRS-2, phospho-phosphatidylinositol 3-kinase (P-PI3K), PI3K, phospho-Akt (P-Akt) (Ser473), Akt, and pancreatic and duodenal homeobox-1 (PDX-1) was measured by Western blot analysis. All antibodies were purchased from Cell Signaling (Boston, MA, USA). The cells were lysed in RIPA buffer (Cell Signaling, Danvers, MA, USA) with a protease inhibitor for 20 min on ice. Samples containing a 20 μg concentration of protein were separated by 10% sodium dodecyl sulfate polyacrylamide gel and then transferred onto polyvinylidene difluoride membranes. The membranes were incubated with primary antibodies against P-IRS-2 (Ser731), IRS-2, P-PI3K, PI3K, P-Akt (Ser473), Akt, PDX-1, and glyceraldehyde 3-phosphate dehydrogenase (GAPDH) overnight at 4 ℃, followed by incubation with horseradish peroxidase (HRP)-conjugated anti-rabbit secondary antibodies at room temperature for 1 h. The expression of proteins was visualized by an enhanced chemiluminescence reagent (GE Healthcare UK Limited, Buckinghamshire, UK) and a chemiluminescence system (FUSION Solo, PEQLAB Biotechnologie GmbH, Erlangen, Germany).

### 4.6. Assay of α-Glucosidase Activity

α-Glucosidase activity was identified according to the manual of the Sigma-Aldrich commercial kits (Art. No. MAK123, St. Louis, MO, USA). AEA (**3**; 20 μL) was mixed with α-glucosidase enzyme solution (200 μL). The mixture was incubated at 37 °C for 20 min and its absorbance at 405 nm was measured using a PowerWave XS microplate reader (Bio-Tek Instruments, Winooski, VT, USA).

### 4.7. Statistical Analysis

Statistical significance was determined using one-way analysis of variance (ANOVA) and multiple comparisons with a Bonferroni correction. P values less than 0.05 indicated statistical significance. All analyses were performed using SPSS Statistics ver. 19.0 (SPSS Inc., Chicago, IL, USA).

## 5. Conclusions

Based on the results, we report that AEA (**3**) from *A. galanga* exerted α-glucosidase inhibitory activity and GSIS effect. In addition, their GSIS effects were supported by increased expressions of IRS-2, PI3K, Akt, and PDX-1. Although further studies are necessary to investigate the safety and effectiveness of AEA (**3**) in animal models of T2D, AEA (**3**) can be further developed into an alternative option for treating T2D.

## Data Availability

Not applicable.

## Supplementary Materials

The following supporting information can be downloaded at: https://www.mdpi.com/article/10.3390/plants12030579/s1, General experimental procedures; Figure S1. ^1^H-NMR spectrum of HCA (1) (500MHz, CDCl_3_); Figure S2. ^1^H-NMR spectrum of ACA (2) (500MHz, CDCl_3_); Figure S3-1. ^1^H-NMR spectrum of AEA (3) (500MHz, CDCl_3_); Figure S3-2. ^13^C-NMR spectrum of AEA (3) (125MHz, CDCl_3_); Figure S4. ^1^H-NMR spectrum of eugenyl acetate (4) (500MHz, CDCl_3_); Figure S5. ^1^H-NMR spectrum of *p*-coumaraldehyde (5) (500MHz, acetone-*d*_6_); Figure S6. ^1^H-NMR spectrum of *p*-acetoxycinammyl alcohol (6) (500MHz, CDCl_3_); Figure S7. ^1^H-NMR spectrum of *p*-coumaryl diacetate (7) (500MHz, CDCl_3_); Table S1. Specific rotation values of compounds 1–3. References [26,28] are cited in the supplementary materials.
