# Supplementary material for: (1′S)-1′-Acetoxyeugenol Acetate Enhances Glucose-Stimulated Insulin Secretion
Source: Plants (Basel). 2023 Jan 28;12(3):579. doi: 10.3390/plants12030579 (PMC9919771; doi:10.3390/plants12030579)
Supplement: Supplementary file 1 [file plants-12-00579-s001.zip › plants-2071173-supplementary.pdf]

(1'*S*)-1'-Acetoxyeugenol Acetate Enhances Glucose-Stimulated Insulin Secretion

Supplementary Materials

Contents

General experimental procedures

NMR data of isolated compounds 1–7

Figure S1. ^{1}H-NMR spectrum of HCA (**1**) (500MHz, $CDCl_3$)

Figure S2. ^{1}H-NMR spectrum of ACA (**2**) (500MHz, $CDCl_3$)

Figure S3-1. ^{1}H-NMR spectrum of AEA (**3**) (500MHz, $CDCl_3$)

Figure S3-2. ^{13}C-NMR spectrum of AEA (**3**) (125MHz, $CDCl_3$)

Figure S4. ^{1}H-NMR spectrum of eugenyl acetate (**4**) (500MHz, $CDCl_3$)

Figure S5. ^{1}H-NMR spectrum of *p*-coumaraldehyde (**5**) (500MHz, acetone-d_6)

Figure S6. ^{1}H-NMR spectrum of *p*-acetoxycinammyl alcohol (**6**) (500MHz, $CDCl_3$)

Figure S7. ^{1}H-NMR spectrum of *p*-coumaryl diacetate (**7**) (500MHz, $CDCl_3$)

Table S1. Specific rotation values of compounds **1–3**

General experimental procedures

To isolate the compounds from *A. galangal* rhizomes, chromatographic methods were performed by Sephadex LH-20 (Merck, MA, USA), Silica gel (Merck, 230-400 mesh and 70-230 mesh, ASTM), and Diaion HP-20 (Mitsubishi Chemical, Tokyo, Japan) resin for open column chromatography. Then, medium pressure liquid chromatography (MPLC) was conducted by COMBIFLASH systems (Teledyne Isco, NE, USA) with pre-packed cartridges—Redi Sep-Silica (12 g, 24 g, 40 g, Teledyne Isco) and Redi Sep-C18 (13 g, 26 g, 43 g, 130 g, Teledyne Isco). Preparative high performance liquid chromatography (prep HPLC) was carried out by Waters purification system (Waters corporation, MA, USA) equipped with 1525 pump, PDA 1996 detector, and Luna NX-C18 100A column (250.0 × 21.2 mm i.d., 10.0 μm, Phenomenex, CA, USA). All fractions were monitored by thin layer chromatography (TLC) analyses using Silica gel 60 F254 (Merck, MA, USA) and RP-18 F254S (Merck, MA, USA) plates. After TLC development in confirmed solvent system, TLC plates were charred by 20% H_2SO_4 (v/v) reagent (Duksan, Seoul, Korea) and then heated at 123 °C for 15 min. To determine the structures of isolated compounds, optical rotations were obtained by a Jasco P-2000 polarimeter (JASCO, Tokyo, Japan), using a 10 mm microcell. A JEOL 500 MHz NMR spectrometer (JEOL, Tokyo, Japan) was used for NMR analysis of compounds.

NMR data of isolated compounds 1–7

Figure S1. ^{1}H-NMR spectrum of HCA (**1**) (500MHz, $CDCl_3$)

^{1}H-NMR δ_H 2.29 (3H, s, H-OCOC$\underline{H_3}$), 5.19 (1H, overlapped, H-1′), 5.19 (1H, overlapped, H-3′a), 5.34 (1H, dt, *J* = 17.0, 1.0 Hz, H-3′b), 6.01 (1H, ddd, *J* = 17.0, 10.0, 6.0 Hz, H-2′), 7.06 (2H, d, *J* = 8.5 Hz, H-2, 6), 7.37 (2H, d, *J* = 8.5 Hz, H-3, 5)

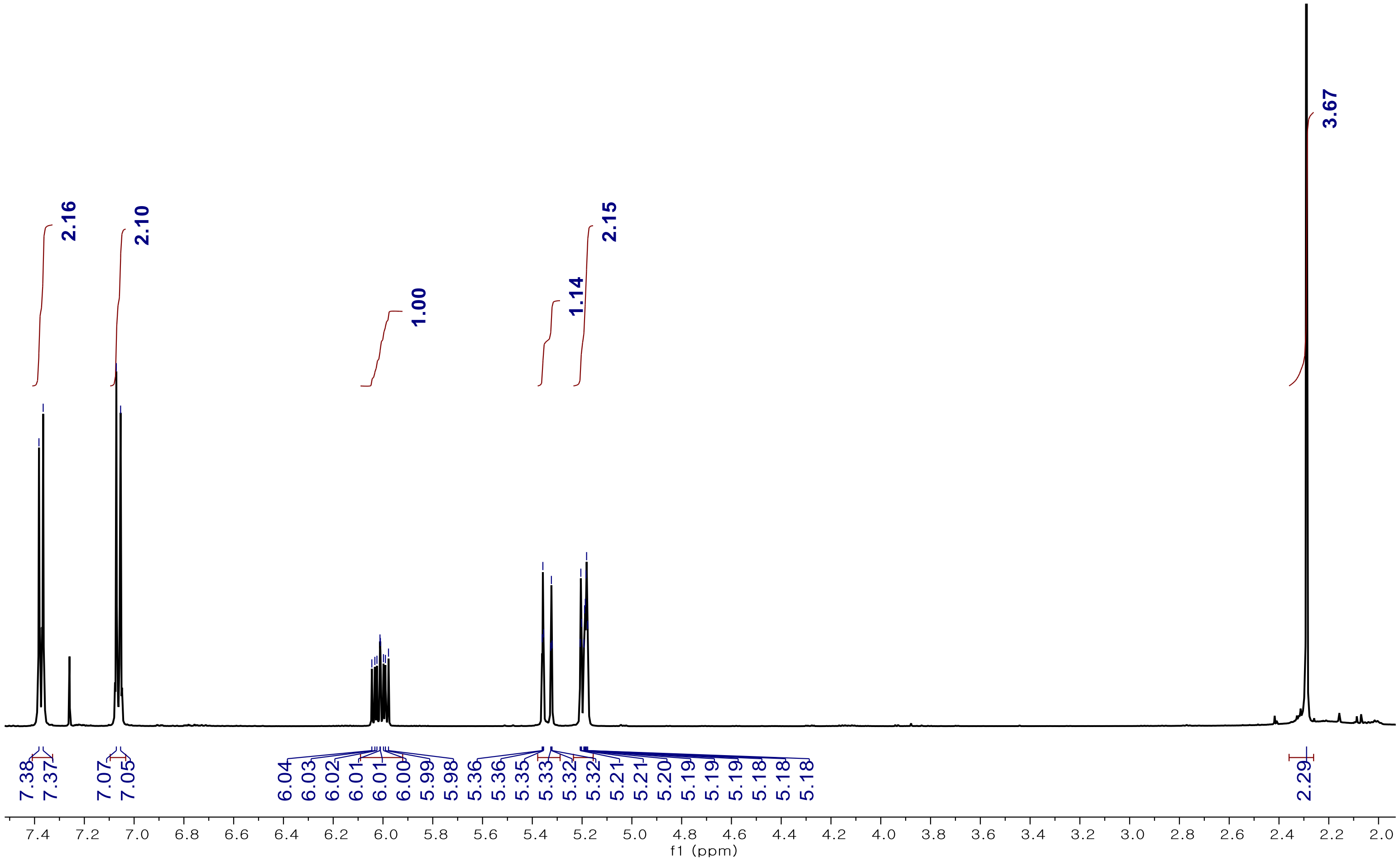

Figure S2. ^{1}H-NMR spectrum of ACA (**2**) (500MHz, $CDCl_3$)

^{1}H-NMR δ_H 2.11 (3H, s, 1′-OCOC<u>H</u>$_3$), 2.30 (3H, s, 4-OCOC<u>H</u>$_3$), 5.25 (1H, dt, *J* = 10.0, 1.0 Hz, H-3′a), 5.30 (1H, dt, *J* = 17.0, 1.0 Hz, H-3′b), 5.98 (1H, ddd, *J* = 17.0, 10.0, 6.0 Hz, H-2′), 6.26 (1H, d, *J* = 6.0, 1.5 Hz, H-1′), 7.07 (2H, d, *J* = 8.5 Hz, H-2, 6), 7.37 (2H, d, *J* = 8.5 Hz, H-3, 5)

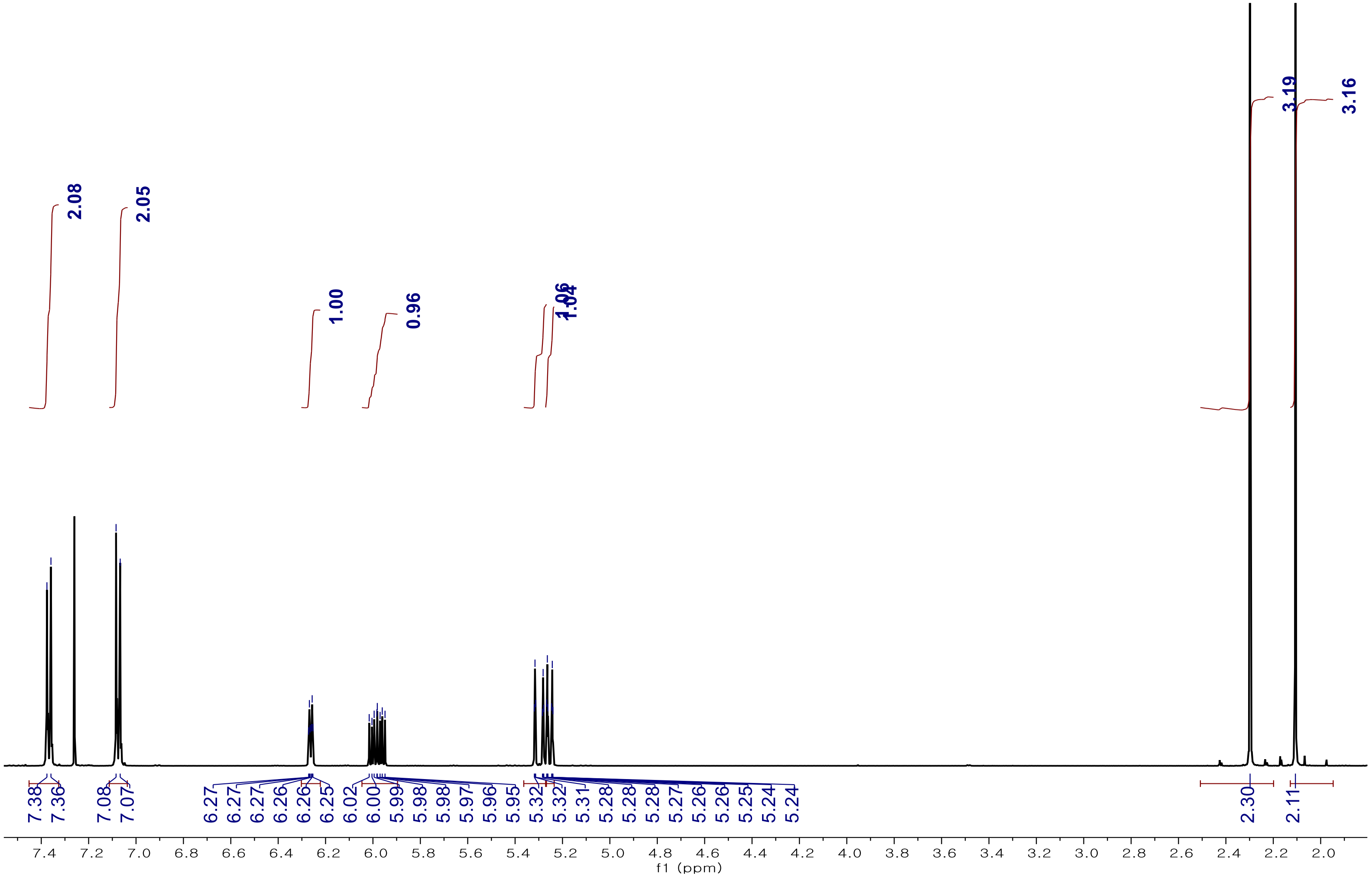

Figure S3-1. ^{1}H-NMR spectrum of AEA (**3**) (500MHz, $CDCl_3$)

^{1}H-NMR δ_H 2.11 (3H, s, 1′-OCOC<u>H</u>$_3$), 2.31 (3H, s, 4-OCOC<u>H</u>$_3$), 3.83 (3H, s, 3-OC<u>H</u>$_3$), 5.26 (1H, dt, *J* = 10.0, 1.5 Hz, H-3′a), 5.31 (1H, dt, *J* = 17.0, 1.5 Hz, H-3′b), 5.98 (1H, ddd, *J* = 17.0, 10.0, 6.0 Hz, H-2′), 6.25 (1H, d, *J* = 6.0, 1.5 Hz, H-1′), 6.94 (1H, dd, *J* = 8.5, 2.0 Hz, H-6), 6.94 (1H, d, *J* = 1.5 Hz, H-2), 7.01 (1H, d, *J* = 8.5 Hz, H-5)

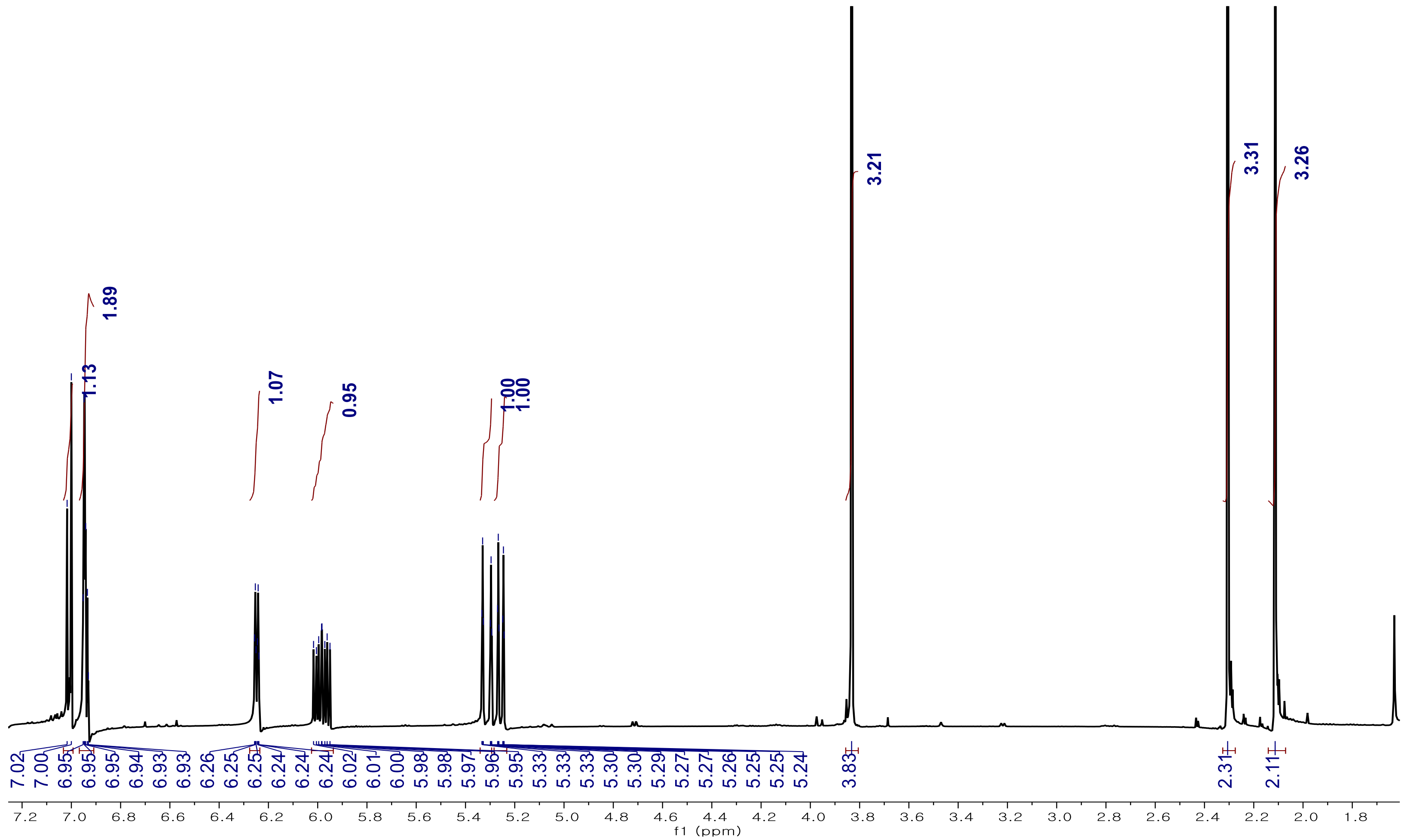

Figure S3-2. ^{13}C-NMR spectrum of AEA (**3**) (125MHz, $CDCl_3$)

^{13}C-NMR (125 MHz, $CDCl_3$) δc 170.1 (OCOCH$_3$), 169.2 (OCOCH$_3$), 151.2 (C-3), 139.7 (C-4), 137.9 (C-1), 136.1 (C-2′), 123.0 (C-5), 119.8 (C-6), 117.2 (C-3′), 111.6 (C-2), 75.9 (C-1′), 56.1 (OCH$_3$), 21.4 (OCOCH$_3$), 20.9 (OCOCH$_3$).

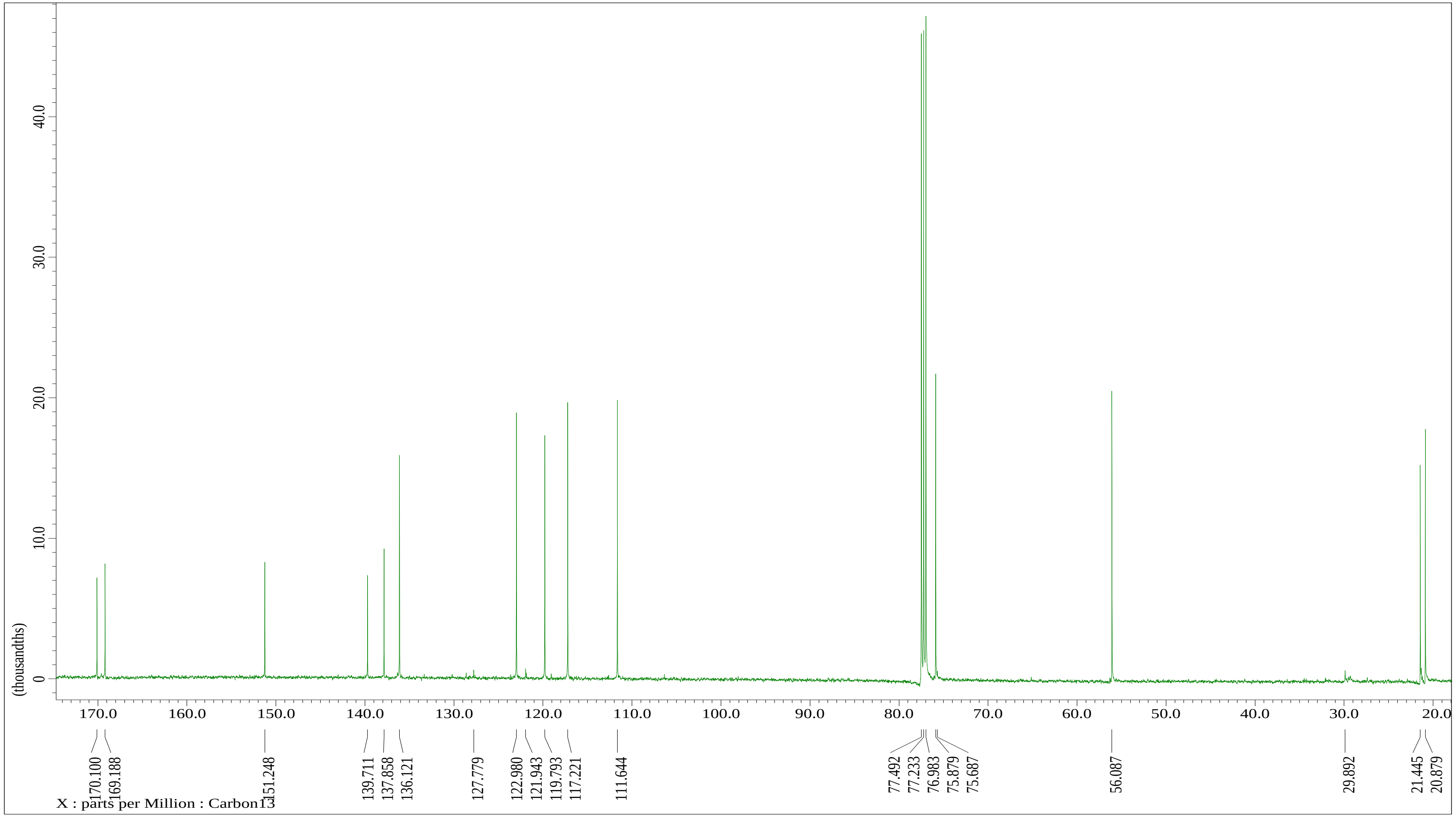

Figure S4. ^{1}H-NMR spectrum of eugenyl acetate (**4**) (500MHz, $CDCl_3$)

^{1}H-NMR δ_H 2.31 (3H, s, 4-OCOC<u>H</u>$_3$), 3.38 (2H, m, H-1′), 3.82 (3H, s, 3-OC<u>H</u>$_3$), 5.07–5.13 (2H, overlapped, H-3′a/H-3′b), 5.96 (1H, ddt, *J* = 17.0, 10.0, 6.0 Hz, H-2′), 6.77 (1H, dd, *J* = 8.0, 2.0 Hz, H-6), 6.79 (1H, d, *J* = 2.0 Hz, H-2), 6.95 (1H, d, *J* = 8.0 Hz, H-5)

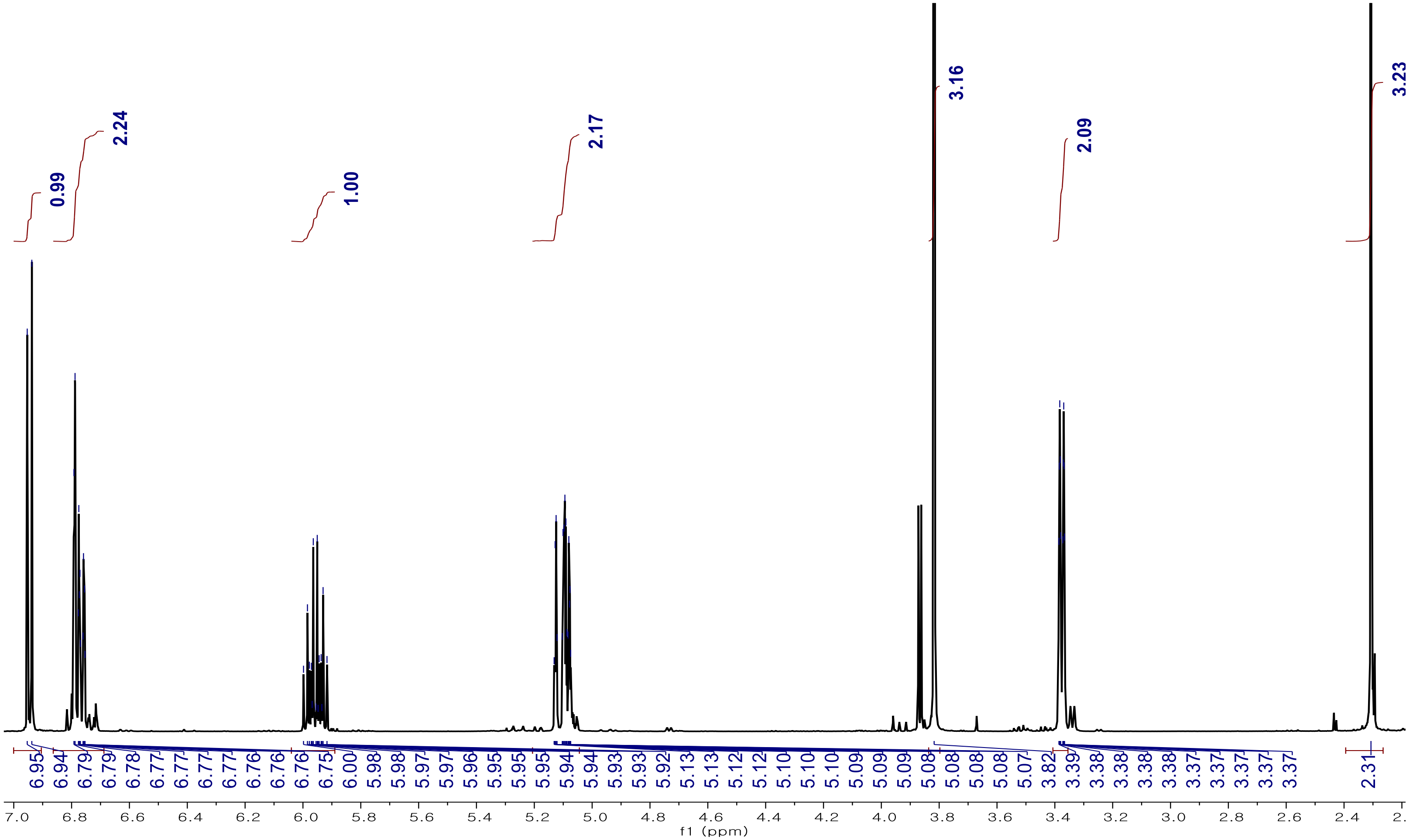

Figure S5. ^{1}H-NMR spectrum of *p*-coumaraldehyde (**5**) (500MHz, acetone-*d*$_6$)

^{1}H-NMR δ_H 6.61 (1H, dd, *J* = 16.0, 7.5 Hz, H-2′), 6.94 (2H, d, *J* = 8.5 Hz, H-2, 6), 7.59 (1H, d, *J* = 16.5 Hz, H-1′), 7.61 (2H, d, *J* = 9.0 Hz, H-3, 5), 9.64 (1H, d, *J* = 7.5 Hz, H-3′)

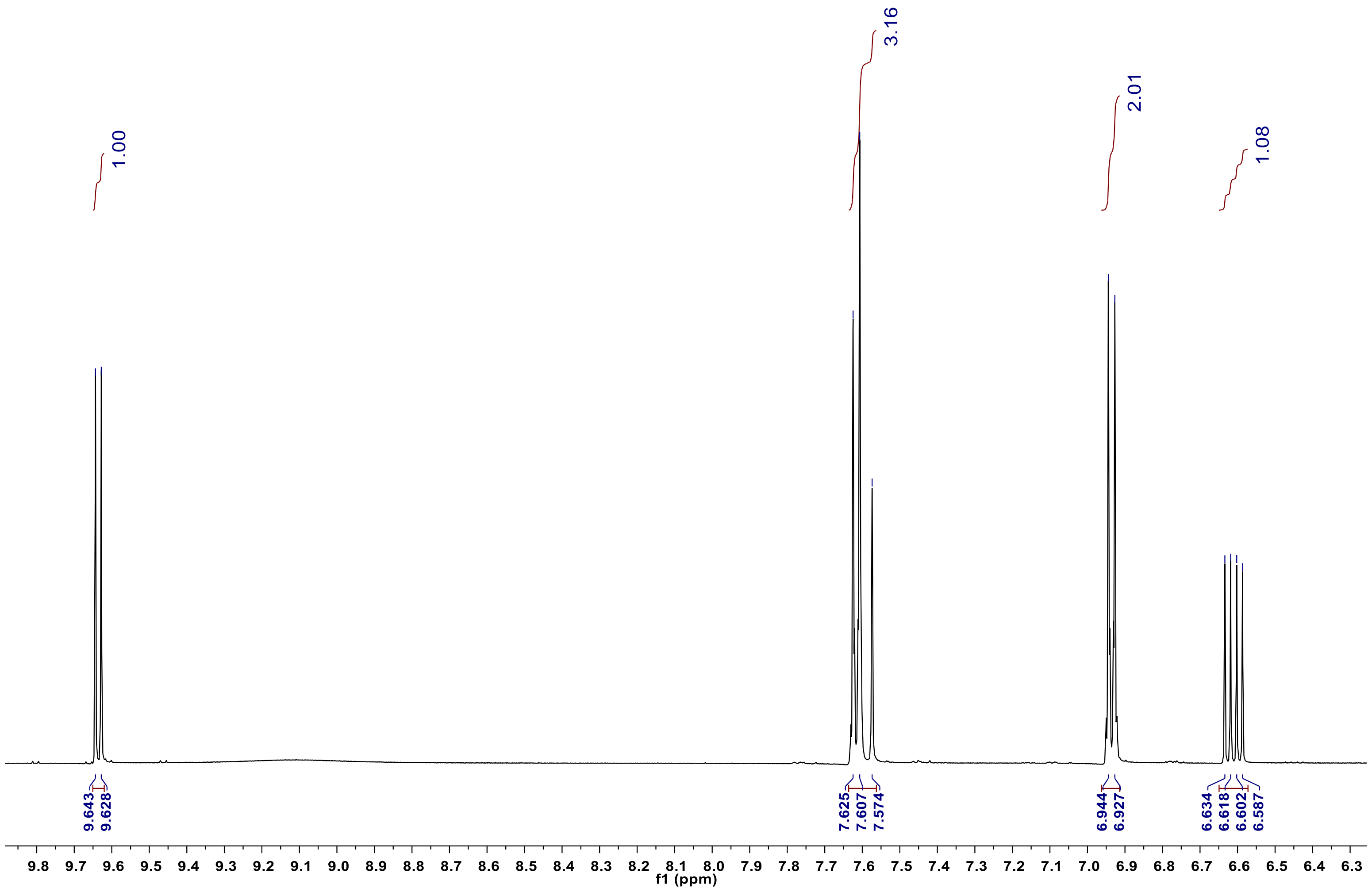

Figure S6. ^{1}H-NMR spectrum of *p*-acetoxycinammyl alcohol (**6**) (500MHz, $CDCl_3$)

^{1}H-NMR δ_H 2.29 (3H, s, 4-OCOC<u>H</u>$_3$), 4.29 (2H, dd, *J* = 5.5, 1.5 Hz, H-3′), 6.29 (1H, dt, *J* = 16.0, 5.5 Hz, H-2′), 6.57 (1H, dt, *J* = 16.0, 1.5 Hz, H-1′), 7.03 (2H, d, *J* = 8.5 Hz, H-2, 6), 7.37 (2H, d, *J* = 8.5 Hz, H-3, 5)

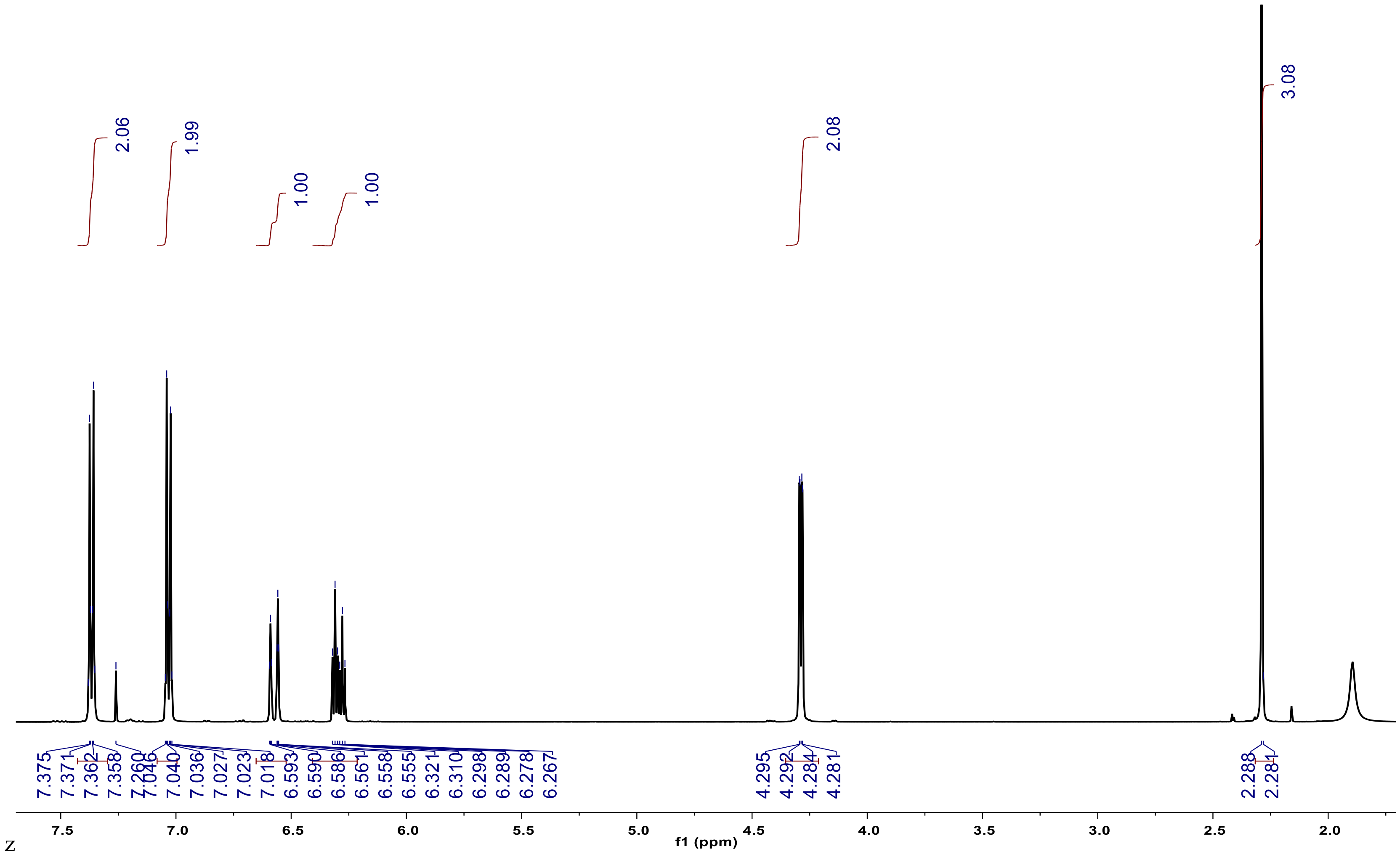

Figure S7. ^{1}H-NMR spectrum of *p*-coumaryl diacetate (**7**) (500MHz, $CDCl_3$)

^{1}H-NMR δ_H 2.10 (3H, s, 3′-$OCOCH_3$), 2.29 (3H, s, 4-OCOC$\underline{H_3}$), 4.71 (2H, dd, *J* =6.5, 1.5 Hz, H-3′), 6.23 (1H, dt, *J* = 16.0, 6.5 Hz, H-2′), 6.63 (1H, dt, *J* = 16.0, 1.5 Hz, H-1′), 7.05 (2H, d, *J* = 8.5 Hz, H-2, 6), 7.39 (2H, d, *J* = 8.5 Hz, H-3, 5)

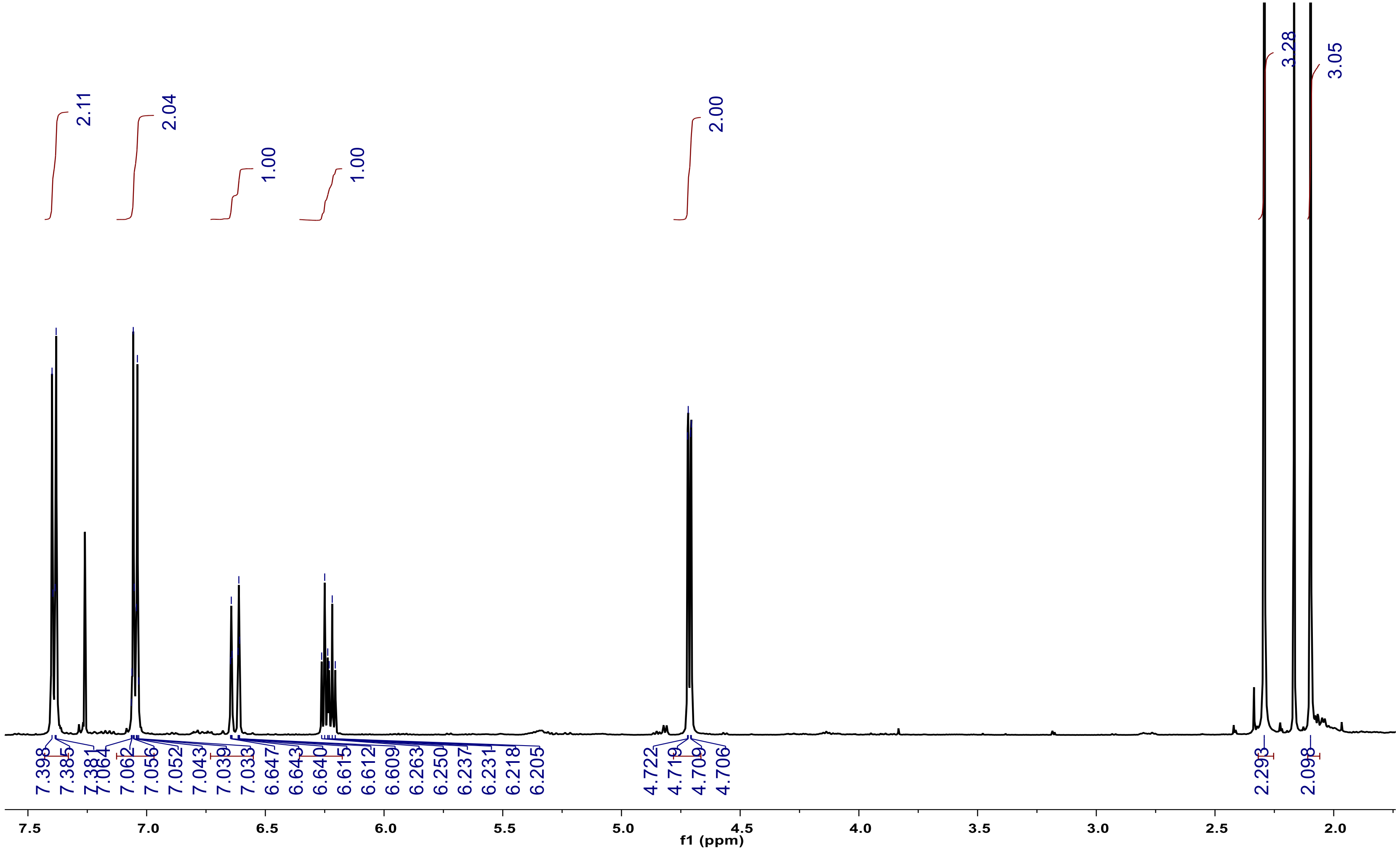

Table S1. Specific rotation values of compounds **1–3**

Compound	$[\alpha]_D^{20}$	Previously reported data		
		**(*S*)-isomer**	**(*R*)-isomer**	**Ref.**
(±)-1′-hydroxychavicol acetate (HCA; **1**)	−0.4° (*c* 0.1, EtOH)	−41° (*c* 0.82, EtOH)	+44° (*c* 0.73, EtOH)	[1]
(1′*S*)-1′-acetoxychavicol acetate (ACA; **2**)	−50.2° (*c* 0.1, EtOH)	−40° (*c* 0.92, EtOH)	+42° (*c* 0.76, EtOH)	[1]
(−)-1′-acetoxyeugenol acetate (AEA; **3**)	−16.6° (*c* 0.21, EtOH)	−17.8° (*c* 0.44, EtOH)	-	[2]

1. Lee, S.-J.; Ando, T., Optically active 1-acetoxychavicol acetate and its positional isomers: synthesis and repellent effect against adzuki bean weevil. *JOURNAL OF PESTICIDE SCIENCE-PESTICIDE SCIENCE SOCIETY OF JAPAN-JAPANESE EDITION-* **2001,** 26, (1), 76-81.
2. Noro, T.; Sekiya, T.; Katoh, M.; ODA, Y.; MIYASE, T.; KUROYANAGI, M.; UENO, A.; FUKUSHIMA, S., Inhibitors of xanthine oxidase from Alpinia galanga. *Chemical and pharmaceutical bulletin* **1988,** 36, (1), 244-248.
